# Serum miRNAs miR-206, 143-3p and 374b-5p as potential biomarkers for amyotrophic lateral sclerosis (ALS)

**DOI:** 10.1016/j.neurobiolaging.2017.03.027

**Published:** 2017-07

**Authors:** Rachel Waller, Emily F. Goodall, Marta Milo, Jonathan Cooper-Knock, Marc Da Costa, Esther Hobson, Mbombe Kazoka, Helen Wollff, Paul R. Heath, Pamela J. Shaw, Janine Kirby

**Affiliations:** a Department of Neuroscience, Sheffield Institute for Translational Neuroscience, University of Sheffield, Sheffield, UK; b Department of Biomedical Science, University of Sheffield, Sheffield, UK

**Keywords:** Amyotrophic lateral sclerosis, Serum, MicroRNA, Polymerase chain reaction, Biomarker

## Abstract

Amyotrophic lateral sclerosis (ALS) is a fatal, neurodegenerative condition characterized by loss of motor neurones and progressive muscle wasting. There is no diagnostic test for ALS therefore robust biomarkers would not only be valuable for diagnosis, but also for the classification of disease subtypes, monitoring responses to drugs and tracking disease progression. As regulators of gene expression, microRNAs (miRNAs) are increasingly used for diagnostic and prognostic purposes in various disease states with increasing exploration in neurodegenerative disorders. We hypothesize that circulating blood-based miRNAs will serve as biomarkers and use miRNA profiling to determine miRNA signatures from the serum of sporadic ALS patients compared to healthy controls and patients with diseases that mimic ALS. A number of differentially expressed miRNAs were identified in each set of patient comparisons. Validation in an additional patient cohort showed that miR-206 and miR-143-3p were increased and miR-374b-5p was decreased compared to controls. A continued change in miRNA expression persisted during disease progression indicating the potential use of these particular miRNAs as longitudinal biomarkers in ALS.

## 1 Introduction

Amyotrophic lateral sclerosis (ALS) is a clinical subtype of motor neurone disease, a fatal, neurodegenerative condition characterized by selective loss of motor neurones and progressive muscle wasting, leading to death within 2–5 years of symptom onset. It is one of the most common adult onset neurodegenerative diseases with a prevalence of 6–8 per 100,000 (Mcdermott and Shaw, 2008). ALS is a complex disease with multiple pathogenic mechanisms having been proposed including: oxidative stress, excitotoxicity, mitochondrial dysfunction, protein aggregation, defective axonal transport, dysregulated endosomal trafficking, neuroinflammation, and dysregulation of RNA processing (reviewed by Ferraiuolo et al. (2011)). The majority of diagnosed ALS cases are sporadic ALS (sALS; 90%) with no obvious family history, however, in recent years several genes have been described in familial ALS, which account for 5%–10% of all ALS cases (Byrne et al., 2011). The most common of these is an intronic hexanucleotide repeat expansion in the gene chromosome 9 open reading frame 72 (*C9orf72*) which has been shown to decrease mRNA levels (Baumer et al., 2014, Dejesus-Hernandez et al., 2011, Renton et al., 2011), and accounts for ∼40% of all familial ALS and ∼7% of sALS cases (Majounie et al., 2012). Other commonly associated ALS genes include mutations in *SOD1* leading to a toxic gain of function and protein aggregation (Kaur et al., 2016) and mutations in *TARDBP* and *FUS* causing dysregulation of RNA processing and protein aggregation (Lattante et al., 2013).

There is no single, pure diagnostic test for ALS, with diagnosis currently dependent upon referral to a neurologist for assessment. There is an average delay of 1 year from symptom onset to a confirmed diagnosis, typically midway through the patient's disease course (Cellura et al., 2012, Zoccolella et al., 2006). In such a rapidly progressive disease this delay is a significant obstacle to potential therapeutic strategies (Cloutier et al., 2015). ALS is clinically heterogeneous, with multiple subtypes associated with different survival times and overt symptoms. Robust biomarkers would be valuable for the initial diagnosis, the classification of various subtypes of disease, monitoring responses to therapeutic agents and tracking disease progression (Turner et al., 2009). In addition, the use of biomarkers may help to establish suitable patient subsets for clinical and therapeutic development trials (Cloutier et al., 2015, Nzwalo et al., 2014, Turner and Benatar, 2015, Turner et al., 2009). Blood is an attractive source of biomarkers interacting with every tissue in the body, and identifying changes in blood mRNA/protein has previously been used to reflect pathological changes in neurodegenerative diseases (Borovecki and Habek, 2010, Sharp et al., 2006). Sample collection is straight-forward, relatively noninvasive, and part of standard clinical practice.

As master regulators of gene expression, microRNAs (miRNAs) are increasingly used for diagnostic and prognostic purposes in various disease states including cancer (Ma and Weinberg, 2008), with increasing application in the field of neurodegenerative disorders. miRNAs are a novel class of small, noncoding RNA molecule predicted to posttranscriptionally regulate at least one third of human genes (Lewis et al., 2005) acting via binding to complementary regions of targeted mRNA (Bartel, 2009). An increasing number of studies have demonstrated the existence of miRNAs in the blood at detectable levels (Chen et al., 2008, Daniels et al., 2014, Sethi et al., 2013). miRNAs from the blood are exceptionally stable, resisting RNase degradation, different storage temperatures, low/high pH conditions, and multiple freeze thaw cycles making them ideal as potential biomarkers (Botta-Orfila et al., 2014, Chen et al., 2008, Jin et al., 2013, Ren et al., 2016). Recently, serum-based miRNA screening identified 24 significantly downregulated miRNAs in premanifesting ALS mutation carriers, up to 2 decades or more before their symptomatic disease onset (Freischmidt et al., 2014). miR-206 was upregulated in serum derived from ALS patients as well as in SOD1^G93A^ mice compared to control mice (Toivonen et al., 2014). In a larger patient cohort, the expression of miR-338-3p was significantly upregulated in the serum of sALS patients, potentially helping in the understanding of sALS pathogenesis and early diagnosis biomarkers (de Felice et al., 2014). Clearly, the role of miRNAs and their potential use as biomarkers in ALS requires further investigation.

In this study we hypothesized that blood based miRNAs will serve as informative biomarkers useful in sALS diagnosis, monitoring of disease progression and/or prognosis prediction. Initial miRNA profiling was used to determine miRNA signatures from the serum of sALS patients compared to healthy controls and patients with diseases that mimic sALS providing a number of differentially expressed miRNAs identified in each set of patient comparisons. Validation of miR-206, miR-143-3p, and miR-374b-5p was completed in an additional sALS patient/control cohort with their expression investigated during disease progression to establish their potential use as longitudinal biomarkers in ALS.

## 2 Material and methods

### 2.1 Discovery study patient cohort

All blood samples were taken in the morning following an overnight fast and collected in serum-separating tubes and stored at 4 °C. Serum was harvested from whole blood within 24 hours of the sample collection and stored at −80 °C until miRNA extraction was completed. All sALS samples were taken at the time of diagnosis before starting on a course of riluzole (Rilutek). The disease mimic group was divided into 5 diagnostic categories as follows:

(1) Noninflammatory neuropathies
(2) Myopathies (including inclusion body myositis and polymyositis)
(3) Inflammatory neuropathies (including Guillain–Barré syndrome and multifocal motor neuropathy)
(4) Structural spinal disorders
(5) Myasthenia gravis

All ALS patients were classified as sALS. The average age at disease onset in the sALS cohort (*n* = 27) was 64 years (range 44–77 years), and the average age of controls (*n* = 25) was 65 years (range 49–77). Details of the full discovery cohort are shown in Table 1.

**Table 1:** Discovery patient cohort

Group	*n*	Age (range)	Gender (M:F)
sALS	27	64 (44–77)	15:12
Control	25	65 (49–77)	12:13
Noninflammatory neuropathy	7	65 (42–77)	5:2
Myopathy	7	66 (48–77)	3:4
Inflammatory neuropathy	8	59 (28–75)	5:3
Structural spinal disorders	6	57 (42–72)	4:3
Myasthenia gravis	8	68 (52–87)	4:3

Key: sALS, sporadic amyotrophic lateral sclerosis.

### 2.2 Validation study patient cohort—Qiagen custom quantitative PCR (qPCR) arrays

For validation of qPCR arrays, blood samples were collected from a further patient cohort. To widen the applicability of the test and to determine if the biomarkers remained valid whether or not the individual had fasted, not all blood samples were fasted. In addition, samples from patients on riluzole were also collected and the possible effect of the treatment determined. All sALS samples were taken at the time of diagnosis or within 3 months. The average age of the validation sALS cohort (*n* = 23) was 66 years (range 39–88 years). The average age of control subjects (*n* = 22) was 62 years (range 41–79) (Table 2). Further patient details are provided in Supplementary Table 1.

**Table 2:** Validation patient cohort

Group	*n*	Age (range)	Gender (M:F)
sALS	23	66 (39–88)	13:10
Control	22	62 (41–79)	11:11

Key: sALS, sporadic amyotrophic lateral sclerosis.

### 2.3 Longitudinal study patient cohort

The longitudinal study patient cohort consisted of 22 sALS patients (6F:16M) where 2 samples at different time points were taken from the same individual. Eleven patients were recruited from the validation patient cohort although an additional 11 new patients were recruited to the longitudinal study. The average age of the sALS cohort was 61 years (range 31–85 years) on first sample collection. Patients did not fast, and some patients (11/22) were already on a prescribed course of riluzole at the time of their first blood sample being taken. All blood samples were collected as previously described. The first patient samples (baseline) were taken within 3 months of diagnosis with the second sample (latest) taken at least 3 months after diagnosis (Table 3).

**Table 3:** Longitudinal study patient cohort identifying gender, disease onset site, riluzole state, age at symptom onset, baseline sample age, and the time between samples

Longitudinal study patient cohort						
Gender	Disease onset site	Riluzole naïve	Age at symptom onset (y)	Baseline sample age (y)	Time since diagnosis to baseline sample (mo)	Time since diagnosis to latest sample (mo)
F	Bulbar	Yes	71.4	75.6	0.9	8.3
M	Lower limb	Yes	41.8	43.9	−0.1	13.3
M	Lower limb	Yes	67.5	78.0	0.0	3.3
F	Lower limb	Yes	84.8	85.8	−0.3	11.9
M	Lower limb	Yes	49.7	51.2	−0.2	10.7
M	Bulbar	Yes	70.5	70.9	−1.1	5.9
M	Upper limb	Yes	29.8	31.5	−0.8	10.9
F	Upper limb	Yes	67.2	68.4	−0.2	11.3
M	Lower limb	Yes	40.2	43.1	−0.6	13.0
M	Mixed	Yes	59.2	59.6	−1.2	11.0
M	Mixed	No	59.3	59.9	−0.2	10.1
F	Upper limb	No	63.2	65.8	2.3	14.0
M	Bulbar	No	59.3	59.9	1.4	17.9
M	Lower limb	No	46.5	48.7	1.4	18.3
M	Bulbar	No	56.5	57.1	3.0	10.6
F	Lower limb	No	53.7	57.0	0.0	12.6
M	Lower limb	No	74.2	75.3	1.4	10.5
F	Upper limb	No	77.0	77.8	1.7	7.4
M	Upper limb	No	70.8	71.2	1.4	8.6
M	Bulbar	No	71.8	72.5	1.9	5.5
M	Upper limb	No	30.4	31.9	1.8	8.1
M	Lower limb	Yes	61.1	62.8	−0.3	7.5

### 2.4 miRNA extraction

miRNA from patient serum samples were extracted using the Circulating Nucleic Acid Isolation Kit (Norgen Biotek) according to manufacturer's protocols using 1 mL serum samples. This method of miRNA extraction provided robust, consistent miRNA levels from serum samples.

### 2.5 miRNA expression profiling—discovery study

The expression level of 750 miRNAs was determined using human miRNA TaqMan Low Density Arrays (TLDAs) (Applied Biosystems) in 27 sALS patients, 25 controls, and 36 ALS mimic patients. Samples were divided into batches of 10 containing 3 sALS patients, 3 controls, and 4 disease mimics for each experimental run to minimize any batch specific effects.

The TLDAs were run on an ABI 7900HT qPCR machine using Sequence Detection System software v2.3 according to the manufacturer's recommended methodology. The NormFinder algorithm was applied to identify the optimal normalization miRNAs from a set of candidates consistently expressed across all samples (Andersen et al., 2004). NormFinder ranks candidate normalizers according to their expression stability, the average of 8 of the most stable and highly expressed miRNAs was then applied to the data set as a normalization factor and used to calculate the delta cycle threshold (CT) value.

### 2.6 Qiagen custom qPCR array selection—validation study

A panel of 27 miRNA candidate markers were used to design custom qPCR arrays from Qiagen, selected by the following criteria: (1) *p*-value <0.05 upon comparison of sALS patients with the control group and (2) miRNAs identified as significantly different (*p* < 0.05) in more than one comparison of sALS patients to disease mimic groups. Validation experiments using miScript custom qPCR arrays were carried out using serum obtained from 23 sALS patients and 22 neurologically normal controls.

#### 2.6.1 Qiagen reverse transcription

Custom miScript miRNA PCR arrays containing the 27 miRNA-specific miScript Primer Assays were used, with 12 patient samples per array being investigated (6 control and 6 sALS). In brief, 5 μL of extracted RNA were used in separate miScript RT reactions (37 °C for 60 minutes, 95 °C for 5 minutes, followed by 4 °C) according to manufacturer's instructions. Samples were diluted 5-fold in RNase-free water prior to preamplification.

#### 2.6.2 Qiagen preamplification and quantification by real-time polymerase chain reaction

5 μL diluted cDNA was preamplified according to the manufacturer's protocol with relevant miScript preamplification reaction components including the relevant miScript PreAMP Primer Mix (custom designed or in house prepared). Cycling conditions were as follows: 95 °C for 15 minutes, 2 cycles of 30 seconds at 94 °C, 60 seconds at 55 °C and 60 seconds at 70 °C followed by 10 cycles of 30 seconds at 94 °C, 3 minutes at 60 °C. Amplified cDNA was diluted 5-fold in RNase-free water prior to being added to the miScript qPCR reaction mix containing 2× QuantiTect SYBR Green PCR master mix, universal primer followed by the addition of sample to the custom miScript miRNA PCR array.

The qPCR arrays were run on a CFX384 BioRad Real-Time PCR System using Bio-RAD CFX Manager software according to the manufacturer's recommended conditions. First, relative expression of mature miRNAs was calculated using the comparative CT (2 − ΔΔCT) method and the running of each plate was assessed by the analysis of internal plate controls. The average of 3 prechosen normalization control miRNAs (miR-17-5p, miR-223-3p, miR-24) was applied to the data set as a normalization factor (per plate) used to calculate the delta CT value.

### 2.7 Longitudinal study

miRNAs significantly different in patients compared to controls were investigated over a period of time with samples taken from patients at diagnosis (baseline, within 3 months) and at a later time point at least 3 months after diagnosis (Table 3). Qiagen miScript-based qPCR was carried out as previously described.

### 2.8 Bioinformatic/statistical analysis

Relative expression of mature miRNAs was calculated using high-throughput qPCR package in R statistical computing environment for the TLDA analysis (Dvinge and Bertone, 2009) or through the comparative CT (2 − ΔΔCT) method for specific miScript validation experiments. Qlucore Omics Explorer program (Qlucore AB, Sweden) was used to visualize the discovery cohort data. For each study, all statistical analysis between 2 comparison groups was carried out using the unpaired 2-tailed *t*-tests. Nonparametric Kruskal–Wallis tests were carried out to test whether statistical relevance was present between the different miRNA expression across the 3 different patient groups (bulbar, upper, and lower limb onset). Where statistical relevance was present a pair wise Mann–Whitney *U*-test was employed to identify where statistical differences were present. All statistical analysis was carried out in GraphPad Prism 6 (GraphPad Prism Inc, USA). *p*-values smaller than 0.05 were considered statistically significant. ^∗^*p* < 0.05; ^∗∗^*p* < 0.01; ^∗∗∗^*p* < 0.001.

## 3 Results

### 3.1 miRNA profiling in discovery cohort

**Fig. 1:**
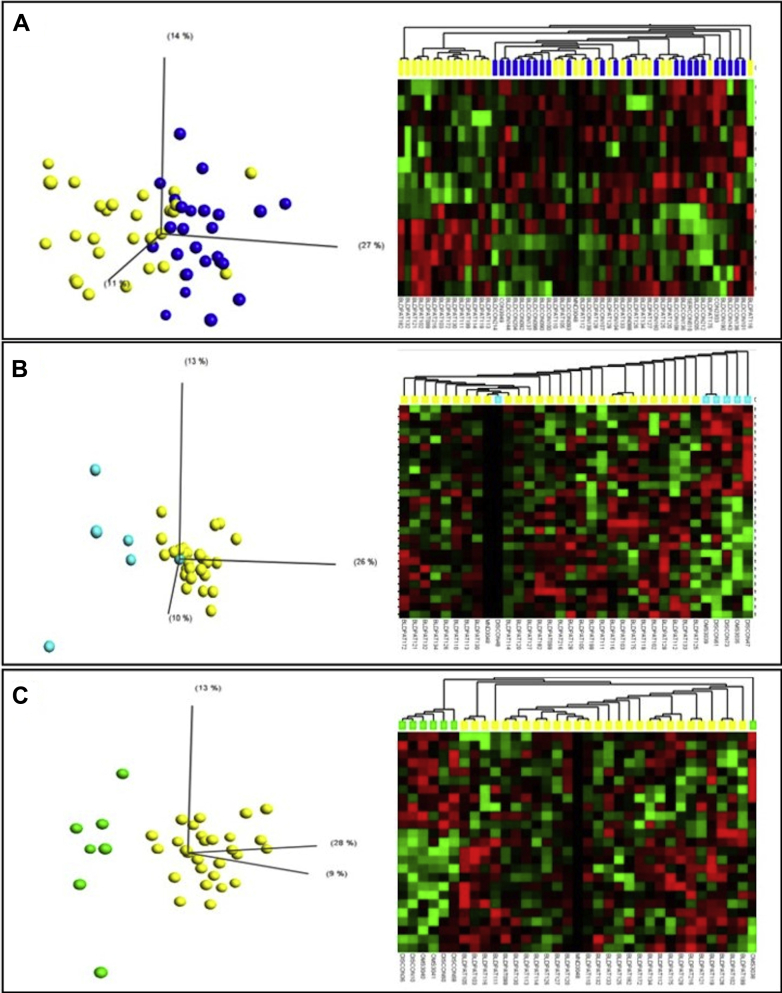
Qlucore output: example PCA plots and heatmaps, *t*-tests *p* ≤ 0.05. (A) ALS (yellow) versus control (blue), (B) ALS versus myopathy (turquoise), (C) ALS versus noninflammatory neuropathy (green). Abbreviations: ALS, amyotrophic lateral sclerosis; PCA, principal component analysis. (For interpretation of the references to color in this figure legend, the reader is referred to the Web version of this article.)

Each patient cohort sample size for each study was determined based on sample availability at the time of testing and correlates with other similar published work (Freischmidt et al., 2013, Toivonen et al., 2014). miRNA profiling was completed in a well-defined discovery cohort of 27 sALS patients, 25 controls, and 36 ALS mimic patients with various neuromuscular conditions. Based on the significantly expressed miRNA there was some overlap between sALS patients and controls (Fig. 1A). However, a clear distinction between sALS and myopathy patients (Fig. 1B) and sALS and noninflammatory neuropathy (Fig. 1C) was apparent. Further distinctions between sALS and other mimic diseases were identified and the number of differentially expressed miRNAs in each set of patient comparisons is shown in Table 4. Comparing miRNA lists revealed differential expression of miRNAs which appeared in more than one comparison between sALS and control/mimic diseases (sALS vs. control group, 12 miRNAs; sALS vs. noninflammatory neuropathy patient group, 5 miRNAs; sALS patient group vs. myopathy patient group, 8 miRNAs; sALS patient group vs. inflammatory neuropathy patient group, 5 miRNAs; sALS patient group vs. structural spinal disorders patient group, 8 mRNAs; sALS patient group vs. myasthenia gravis patient group, 4 miRNAs) suggesting an increased likelihood for them to be sALS specific (Table 5). These miRNAs were taken forward for validation in an additional patient cohort. In total, 27 miRNA formed the basis of the panel used for validation studies with 3 normalization controls, miRNAs, miR-17-5p (0.008), miR-223-3p (0.013), and miR-24 (0.004), which were identified as the top 3 ranked miRNAs stably expressed in all the comparisons made according to NormFinder.

**Table 4:** Differentially expressed miRNAs in multiple patient comparisons

Comparison	Number of cases	Number of miRNA	Average *p*-value
ALS v Con	27 v 25	14	0.0296
ALS v Inflam	27 v 8	17	0.0302
ALS v MG	27 v 8	14	0.0176
ALS v Myop	27 v 7	28	0.0242
ALS v NonInflam	27 v 7	25	0.0222
ALS v Struct	27 v 6	16	0.0303
Con v Inflam	25 v 7	13	0.0220
Con v MG	25 v 8	39	0.0229
Con v Myop	25 v 7	26	0.0243
Con v NonInflam	25 v 7	18	0.0264
Con v Struct	25 v 6	20	0.0277
Inflam v MG	8 v 8	41	0.0226
Inflam v NonInflam	8 v 7	24	0.0217
Inflam v Struct	8 v 6	13	0.0237
Inflam v Myop	8 v 7	13	0.0310
MG v Myop	8 v 7	13	0.0264
MG v NonInflam	8 v 7	22	0.0269
MG v Struct	8 v 6	21	0.0257
Myop v NonInflam	7 v 7	14	0.0255
Myop v Struct	7 v 6	11	0.0248
Noninflam v Struct	7 v 6	14	0.0297

Differentially expressed miRNAs are defined as those having a *p*-value ≤0.05 between the 2 comparable patient groups.

Key: ALS, amyotrophic lateral sclerosis patient group; Con, control subject group; Inflam, inflammatory neuropathy patient group; MG, myasthenia gravis patient group; miRNA, microRNA; Myop, myopathy patient group; NonInflam, noninflammatory neuropathy patient group; Struct, structural spinal disorders patient group; v, versus.

**Table 5:** Individual miRNAs and their significance across the different disease group comparisons in the discovery study

miRNA	Significant sALS comparisons (*p* value)					
	ALS v Con	ALS v Non Inflam	ALS v Struct	ALS v Myop	ALS v Inflam	ALS v MG
hsa-miR-133a-3p	0.021	0.010				
hsa-miR-135b-5p	0.045					
hsa-miR-143-3p	0.021	0.030	0.037	0.008		
hsa-miR-144-3p	0.014	0.014	0.023			
hsa-miR-146b-3p	0.042					
hsa-miR-206	0.000					
hsa-miR-20a-3p	0.003			0.008		
hsa-miR-214-3p	0.041					
hsa-miR-331-3p	0.033					
hsa-miR-374b-5p	0.025					
hsa-miR-518d-3p	0.035					
hsa-miR-551b-3p	0.049					
hsa-let-7d-5p			0.018			0.011
hsa-miR-106b-5p		0.031	0.033			
hsa-miR-133b			0.020		0.020	
hsa-miR-134-5p		0.034		0.004		
hsa-miR-145-3p			0.002		0.020	
hsa-miR-15b-5p		0.021				0.045
hsa-miR-190a-5p		0.019		0.026		
hsa-miR-196b-5p				0.008	0.040	
hsa-miR-301a-3p		0.017		0.018		
hsa-miR-335-5p			0.049	0.028		0.014
hsa-miR-381-3p				0.050		0.020
hsa-miR-500a-3p			0.023	0.032		
hsa-miR-532-3p			0.045	0.048		
hsa-miR-744-5p			0.029		0.039	

miRNA	Significant comparisons (*p* value)													
	Con v Non Inflam	Con v Struct	Con v Myop	Con v Inflam	Con v MG	Non Inflam v Struct	Inflam v Non Inflam	Inflam v Struct	Inflam v Myop	Inflam v MG	Myop v Non Inflam	Myop v Struct	MG v Struct	MG v Myop
hsa-miR-133a-3p													0.028	
hsa-miR-135b-5p					0.011					0.029				
hsa-miR-144-3p		0.041												
hsa-miR-206			0.002											
hsa-miR-20a-3p	0.017													0.048
hsa-miR-214-3p		0.017												
hsa-miR-331-3p			0.024											
hsa-miR-374b-5p				0.005										
hsa-miR-1								0.012				0.008	0.039	
hsa-let-7d-5p		0.049						0.022					0.017	
hsa-miR-106b-5p							0.021	0.023						
hsa-miR-133b			0.025											
hsa-miR-134-5p						0.030						0.032	0.043	
hsa-miR-145-3p						0.010	0.029						0.022	
hsa-miR-15b-5p														
hsa-miR-190a-5p			0.026			0.013	0.003		0.049	0.016			0.043	
hsa-miR-196b-5p			0.000								0.000	0.020		0.013
hsa-miR-301a-3p			0.024	0.020							0.039	0.045		0.032
hsa-miR-335-5p				0.043	0.000					0.046	0.028		0.002	0.016
hsa-miR-381-3p					0.034									

For each miRNA the number of comparisons is shown and the significance of each.

Key: ALS, amyotrophic lateral sclerosis patient group; Con, control subject group; Inflam, inflammatory neuropathy patient group; MG, myasthenia gravis patient group; miRNA, microRNA; Myop, myopathy patient group; n/a, data not applicable; NonInflam, noninflammatory neuropathy patient group; Struct, structural spinal disorders patient group; v, versus.

### 3.2 Validation of miRNA biomarkers in serum

**Fig. 2:**
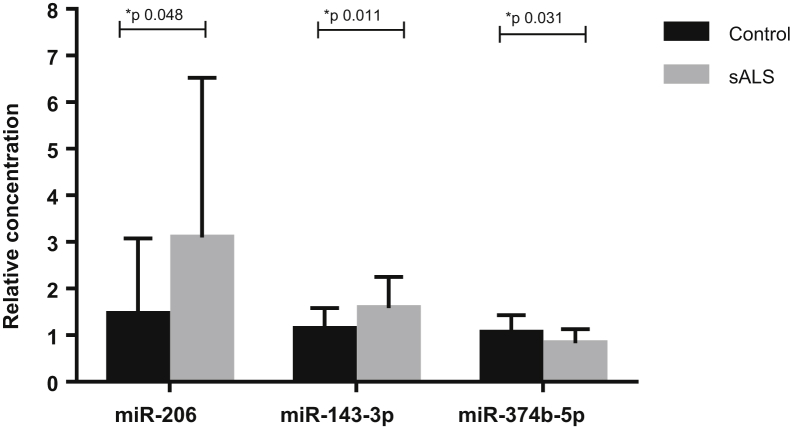
Significant miRNA expression in sALS (*n* = 23) versus control (*n* = 22) subjects as confirmed by qPCR. Unpaired *t*-test, error bars represent SD. Abbreviations: miRNA, microRNA; sALS, sporadic amyotrophic lateral sclerosis; SD, standard deviation.

Of the 12 miRNAs differentially expressed between sALS and control subjects in the discovery cohort, the validation studies found 3 of them; miR-206, miR-143-3p, and miR-374b-5p to be significantly differentially expressed in an additional patient cohort comparing 23 sALS and 22 control subjects (Fig. 2). miR-206 and miR-143-3p were significantly increased in sALS patients compared to controls, whereas miR-374b-5p was significantly decreased in the same comparison. Analysis was carried out to investigate the expression of the 3 candidate miRNAs in the 3 patient subgroups according to site of disease onset (bulbar *n* = 9, upper *n* = 7, and lower limb *n* = 7 onset). No significant difference in miR-206 (*p* = 0.795), miR-143-3p (*p* = 0.245), or miR-374b-5p (*p* = 0.537) between the patient groups was identified.

### 3.3 Riluzole treatment does not affect miRNA expression

**Fig. 3:**
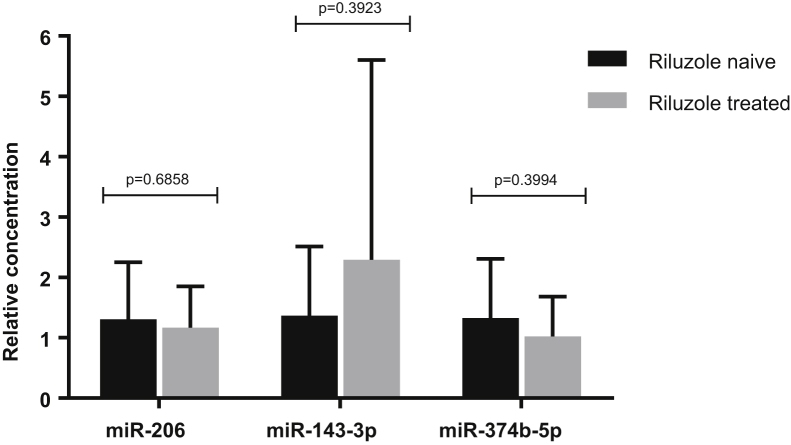
Riluzole versus riluzole naïve patients. miRNA expression (miR-206, miR-143-3p and miR-374b-5p) in riluzole naïve (*n* = 10) and riluzole treated patients (*n* = 13) confirmed by qPCR. Unpaired *t*-test, error bars represent SD. Abbreviations: miRNA, microRNA; SD, standard deviation.

No significant difference in expression of miR-206, miR-143-3p and miR-374b-5p was identified between the 2 patient groups (riluzole naïve [*n* = 13] and riluzole treated [*n* = 10]), demonstrating that riluzole has no effect on the expression of these 3 specific miRNAs in serum (Fig. 3). Further analysis was completed to examine the expression of the other 24 candidate miRNA investigated in the validation study to identify whether riluzole was affecting miRNA expression levels on the whole when comparing sALS patients and controls. When comparing riluzole naïve patients (*n* = 10) to controls (*n* = 22), 2 additional significantly expressed miRNAs were identified; miR-744-5p (*p*-value 0.035) and miR-134-5p (*p*-value 0.015) which were not differentially expressed when comparing patients prescribed riluzole to controls. However, when comparing patients prescribed riluzole (*n* = 13) to controls (*n* = 22), 2 additional significantly expressed miRNAs were identified, miR-381-3p (*p*-value 0.035) and miR-146b-3p (*p*-value 0.029), which were not differentially expressed when comparing riluzole naïve patients compared to controls.

### 3.4 miR-143-3p and miR-374b-5p are consistently changed from diagnosis throughout sALS patients disease course

**Fig. 4:**
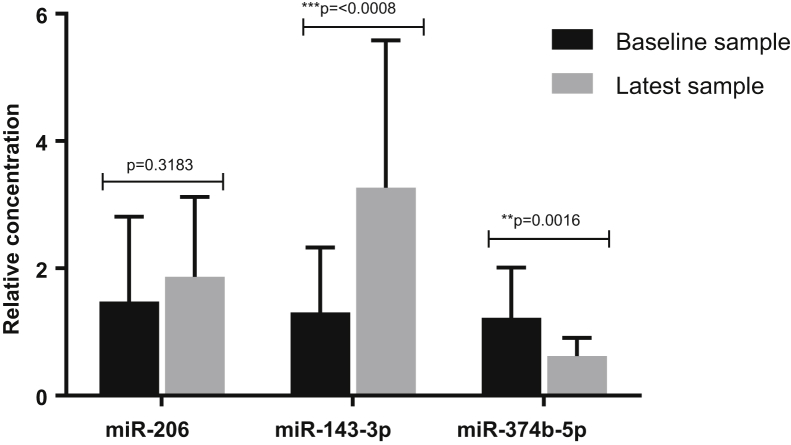
miRNA expression changes over time as confirmed by qPCR in 21 sALS patients. Unpaired *t*-test, error bars represent SD. Abbreviations: miRNA, microRNA; sALS, sporadic amyotrophic lateral sclerosis; SD, standard deviation.

Having established that riluzole does not appear to have a significant effect on miR-206, miR-143-3p, and miR-374b-5p expression, the 2 patient groups (riluzole naïve and riluzole treated) were combined and the analysis of the candidate miRNAs over time was investigated. Results showed a significant increase in miR-143-3p in serum samples taken from sALS patients later in their disease (>3 months) compared to serum samples taken from patients at diagnosis. By contrast miR-374b-5p was significantly decreased in serum samples taken from sALS patients later in their disease (>3 months) compared to serum samples taken from patients at diagnosis (Fig. 4). Whilst miR-206 was increased over progression of the disease, the change over time did not reach significance. Additionally, no significant difference was seen in the expression of the 3 miRNAs in the group of 11 riluzole naïve patients of the longitudinal study compared with their subsequent later sample where patients were prescribed riluzole (data not shown).

**Fig. 5:**
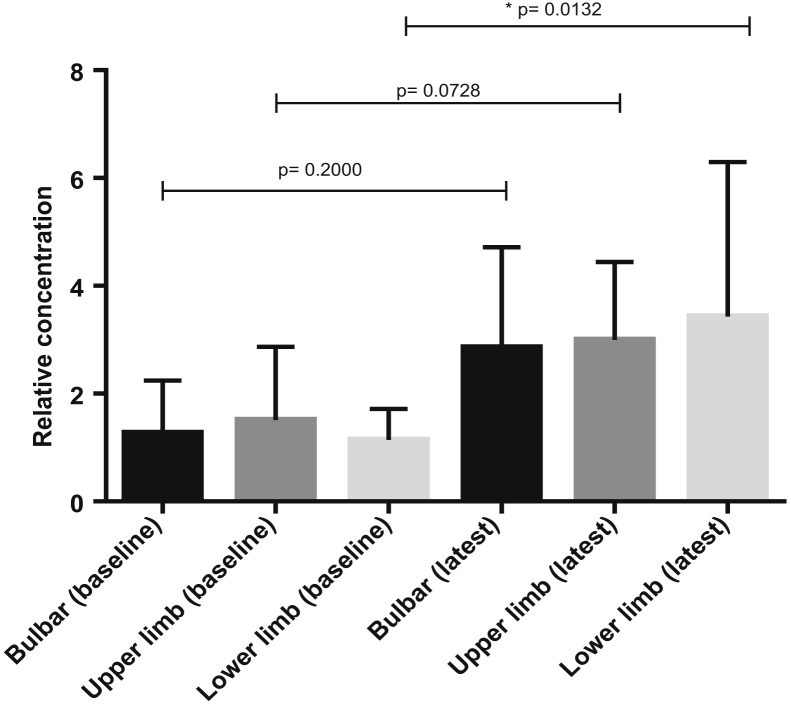
Comparing miR-143-3p expression changes over time in 3 patient groups as confirmed by qPCR in bulbar (*n* = 5), upper (*n* = 6), and lower limb (*n* = 9) onset sALS patients. Mann–Whitney *U* test, error bars represent SD. Abbreviations: sALS, sporadic amyotrophic lateral sclerosis; SD, standard deviation.

Furthermore analysis was carried out to investigate the expression of the 3 candidate miRNAs in the 3 patient subgroups, divided according to site of disease onset (bulbar *n* = 5, upper *n* = 6, and lower limb *n* = 9 onset). Only levels of miR-143-3p were identified as significantly different across the 3 subgroups (miR-143-3p, *p* = 0.042, Kruskal–Wallis test). A significant increase in miR-143-3p was determined in the later sample of lower limb onset patients, while there was a nonsignificant increase in miR-143-3p seen in the later samples of those patients with bulbar and upper limb onset (Fig. 5).

## 4 Discussion

The main findings of this study are; (1) miR-206, miR-143-3p, and miR-374b-5p are differentially expressed in the serum of sALS patients compared to control subjects; (2) the drug riluzole has no effect on the expression of the 3 validated miRNAs in the serum of sALS patients; and (3) miR-143-3p is significantly increased while miR-374b-5p is significantly decreased in the serum of sALS patients over time identifying a continuingly altered miRNA profile associated with disease progression.

Identifying serum-based miRNAs as potential biomarkers could increase our understanding of ALS and this approach has been utilized in developing the understanding of many other neurodegenerative diseases (Ding et al., 2016, Jia and Liu, 2016, Wu et al., 2015). Serum collection comes from a routine, noninvasive blood test making it an ‘easier’ sample to obtain from patients. Several studies have identified potential biomarkers in serum from ALS patients but this is the first longitudinal study investigating miRNA expression levels over time associated with disease progression in human subjects.

Previous animal work has identified an increased expression of miR-206 over time in the ALS SOD1 G93A mouse model (Toivonen et al., 2014) and the murine spinal muscular atrophy model (Valsecchi et al., 2015), correlating with increasing pathology and associated muscle denervation. However, the expression of miR-206 presented in this current study did not identify a significant change over time with human disease progression, yet did show an increased expression of this miRNA in sALS patients compared to controls. This supports human work carried out by Toivonen et al. (2014) where samples were however, taken at only a single time point.

Despite these differences relating to miR-206 expression, the current study does identify an increase in miR-143-3p and decrease in miR-374b-5p over time, with serum samples taken from individual sALS patients at diagnosis and at a later time point. It would appear that such miRNA expression data could be utilized to monitor disease progression and ultimately, through miRNA target analysis, therapeutics could potentially be developed where miR-143-3p and miR-374b-5p are used as biomarkers to assess treatment efficacy and potentially disease prognosis.

During development or as a mature miRNA, miR-143-3p has previously been identified to bind to TDP-43 in vitro (Freischmidt et al., 2013). In contrast to our current findings, Freischmidt reported miR-143-3p as significantly downregulated in serum from a subset of sALS patients. This difference could result from alternative RNA isolation and qPCR protocols used relative to our current study (Freischmidt et al., 2013, Hammerle-Fickinger et al., 2010). In addition, specific information on patient sample collection was not disclosed which might explain the difference in expression between the studies (Freischmidt et al., 2013).

As described for miR-206, miR-143-3p has been implicated with muscle changes. Investigations carried out on an immortalized mouse myoblast cell line (C2C12 cell line) demonstrated that an upregulation of miR-143-3p was negatively associated with myoblast cell differentiation suggesting that miR-143-3p may supress myotube differentiation and maturation. Furthermore it has been proposed that inhibiting miR-143-3p may have beneficial effects in muscle wasting diseases (Du et al., 2016). The longitudinal increased expression of miR-143-3p in the serum of sALS patients may correlate with increasing denervation of muscle during disease progression. In addition, an alternative study carried out on the same cell line identified that over-expression of miR-374b impaired C2C12 cell differentiation, while inhibiting miR-374b expression by 2′-O-methyl antisense oligonucleotides, promoted C2C12 cell differentiation (Ma et al., 2015). In the current study miR-374b-5p is reduced in patient serum over time. This could be a compensatory effect to the degeneration of muscle in ALS and an attempt to restore a balance and support muscle regeneration by promoting myoblast differentiation.

As both miR-143-3p and miR-374b-5p show opposing expression profiles in processes linked to cell differentiation, the previous cell culture work, in combination with the current study, provide an explanation as to the possible consequences of the opposing miRNA expression over time in ALS. Decreasing expression of miR-374b-5p may promote myoblast differentiation to compensate for the muscle degeneration associated with ALS and the increased expression of miR-143-3p over time reflecting progressive muscle denervation associated with disease progression.

The relationship between these validated miRNA with muscle has also been described in a study exploring the association between endothelial cells and smooth muscle cells with miR-143-3p seen as part of the process (Wang et al., 2016). Artificially overexpressing miR-143/145 was seen to alter the levels of metabolites involved in energy production, DNA methylation, and oxidative stress, all processes involved in the pathobiology ALS (Wang et al., 2016).

Selection of miRNAs for validation was based on the discovery work carried out in the initial stage of the current study. Twelve miRNAs were selected for validation because of their significance in sALS versus control subjects while the other 14 miRNAs were chosen based on their differential expression in sALS versus “mimic” diseases. The validation study concentrated on investigating sALS compared to control subjects using the 3 validated miRNAs from the original discovery study patient cohort. Another 9 differentially expressed miRNAs identified were not validated in the additional patient cohort. There are a number of potential explanations for this. As an overall function of miRNAs is to generate rapid and reversible responses their expression is inevitably influenced by outside stresses beyond our control such as the diet and lifestyle choices of the patients. For example, changes in miRNA expression and function as a result of specific dietary compounds such as amino acids, carbohydrates, fatty acids, and vitamins have been reported (Garcia-Segura et al., 2013). Patient recruitment criteria were slightly different for the validation patient cohort compared with the original discovery study, with samples not necessarily being taken following overnight fasting which may account for the lack of validation of some miRNAs. However, where possible conditions were kept the same for patient sampling between each cohort, including samples being taken within 3 months of diagnosis for both the discovery and validation cohorts.

We demonstrated in the current study that riluzole did not have an effect on the expression of the 3 validated miRNA in our patient samples. This is the first study to investigate the potential effect of riluzole on miRNA expression levels in sALS patients. However, it has to be acknowledged that there are further considerations concerning the potential use of miRNA as biomarkers for diseases such as ALS including the recognition that changes in the expression of circulating miRNAs may result from factors unrelated to the disease state including variations in nutrition, medication, and environmental factors (Castro-Villegas et al., 2015, Summerer et al., 2013, Wang et al., 2009).

## 5 Conclusions

In summary, the current study, for the first time, demonstrates the potential use of serum-based miRNAs as biomarkers of ALS disease progression. Importantly this study suggests that an alteration in circulating serum miRNAs in ALS may result from muscle denervation and degeneration and therefore measuring these levels throughout a patient's disease course could prove useful in monitoring disease progression and therapeutic responses.

## Disclosure statement

P. J. S. is a National Institute for Health (NIHR) senior investigator. The remaining authors disclose no conflicts.
