# Supplementary material for: Serum miRNAs miR-206, 143-3p and 374b-5p as potential biomarkers for amyotrophic lateral sclerosis (ALS)
Source: Neurobiol Aging. 2017 Jul;55:123–31. doi: 10.1016/j.neurobiolaging.2017.03.027 (PMC5455071; doi:10.1016/j.neurobiolaging.2017.03.027)
Supplement: Supplementary Table 1 [file mmc1.docx]

|  | **Validation sALS patient cohort** | | | | | | |
| --- | --- | --- | --- | --- | --- | --- | --- |
|  | **Gender** | **Disease onset site** | **Riluzole naïve** | **Age at symptom onset (years)** | **Age at sample taken (years)** | **Diagnostic delay (months)** | **Time since diagnosis to sample taken (months)** |
| **Plate 1** | M | Bulbar | Yes | 75.1 | 76.2 | 13.0 | 0.0 |
|  | M | Bulbar | No | 59.3 | 59.9 | 5.8 | 1.4 |
|  | M | Bulbar | No | 56.5 | 57.1 | 4.5 | 3.0 |
|  | M | Lower limb | Yes | 74.2 | 75.3 | 11.6 | 1.4 |
|  | F | Upper limb | No | 63.2 | 65.8 | 28.9 | 2.3 |
|  | F | Upper limb | No | 53.9 | 54.4 | 3.9 | 1.4 |
| **Plate 2** | M | Lower limb | No | 46.5 | 48.7 | 25.0 | 1.4 |
|  | M | Bulbar | No | 61.8 | 62.3 | 3.9 | 1.2 |
|  | M | Upper limb | No | 70.8 | 71.2 | 3.7 | 1.4 |
|  | F | Upper limb | No | 77.0 | 77.8 | 7.4 | 1.7 |
|  | F | Bulbar | No | 87.6 | 88.7 | 11.9 | 1.3 |
| **Plate 3** | M | Bulbar | No | 71.8 | 72.5 | 6.2 | 1.9 |
|  | M | Upper limb | Yes | 71.5 | 72.4 | 11.2 | -0.8 |
|  | M | Lower limb | Yes | 49.7 | 51.2 | 19.1 | -0.2 |
|  | F | Bulbar | No | 74.5 | 75.3 | 9.0 | 1.1 |
|  | F | Lower limb | Yes | 58.7 | 59.6 | 11.9 | -0.5 |
|  | F | Upper limb | Yes | 67.2 | 68.4 | 15.0 | -0.2 |
| **Plate 4** | F | Lower limb | Yes | 36.8 | 39.0 | 26.9 | -0.2 |
|  | F | Upper limb | No | 80.4 | 81.0 | 9.3 | 0.0 |
|  | F | Bulbar | Yes | 47.7 | 49.2 | 18.0 | -0.5 |
|  | M | Lower limb | No | 77.5 | 78.5 | 14.0 | -1.0 |
|  | M | Bulbar | Yes | 70.5 | 70.9 | 5.2 | -1.1 |
|  | M | Bulbar | Yes | 81.5 | 83.0 | 18.5 | 0.0 |

| **Validation control subject cohort** | | |
| --- | --- | --- |
|  | **Gender** | **Age at sample taken (years)** |
| **Plate 1** | M | 65.6 |
|  | M | 77.7 |
|  | M | 62.7 |
|  | M | 58.6 |
|  | F | 57.0 |
|  | F | 69.6 |
| **Plate 2** | F | 55.8 |
|  | F | 60.6 |
|  | F | 70.8 |
|  | M | 76.4 |
|  | M | 59.6 |
|  | M | 60.0 |
| **Plate 3** | F | 47.5 |
|  | M | 58.4 |
|  | F | 57.2 |
|  | M | 79.8 |
|  | F | 68.9 |
| **Plate 4** | F | 41.4 |
|  | F | 66.9 |
|  | F | 67.4 |
|  | M | 70.8 |
|  | M | 51.4 |

**Supplementary Table 1:** Validation cohort - full patient/subject details.
